# Purulent Skin and Soft Tissue Infections, Challenging the Practice of Incision and Drainage: A Scoping Review

**DOI:** 10.1155/2023/5849141

**Published:** 2023-10-06

**Authors:** Liam Stout, Melanie Stephens, Farina Hashmi

**Affiliations:** ^1^University of Salford, Salford, UK; ^2^Calderdale, and Huddersfield NHS Trust, Huddersfield, UK

## Abstract

**Aim:**

To generate a landscape of the current knowledge in the interventional management and outcomes of purulent skin and soft tissue infections.

**Design:**

This study is a scoping review.

**Methods:**

Electronic searches were undertaken using CINAHL, Medline, Cochrane Library, British Nursing Index, Science Direct, the National Health Service knowledge and library hub, ClinicalTrials.gov, and MedNar. The population, concept, context framework, and the Preferred Reporting Items for Systematic Reviews and Meta-Analyses extension for Scoping Reviews were utilised, supporting a rigorous appraisal and synthesis of literature. *Data Sources*. The initial search and synthesis of literature were completed in January 2022 with repeat searches completed in March 2022 and July 2023. There were no imposed chronological parameters placed on the returned literature.

**Results:**

Nineteen papers were reviewed. Incision and drainage with primary closure, needle aspiration, loop drainage, catheter drainage, and suction drainage are viable adjuncts or alternatives to the traditional surgical management of skin and soft tissue abscesses.

**Conclusion:**

Despite the empirically favourable alternatives to the incision and drainage technique demonstrated, this does not appear to be driving a change in clinical practice. Future research must now look to mixed and qualitative evidence to understand the causative mechanisms of incision and drainage and its ritualistic practice. *Implications*. Ritual surgical practices must be challenged if nurses are to improve the treatment and management of this patient group. This will lead to further practice innovation. *Impact*: This study explored the challenges posed to patients, clinicians, nurses, and stakeholders, resulting from the ritualistic practice of the incision and drainage technique in purulent skin or soft tissue abscesses. Empirically and holistically viable alternatives were identified, impacting all identified entities and recommending a wider holistic study. *Reporting Method*. Adherence to EQUATOR guidance was achieved through the Preferred Reporting Items for Systematic Reviews and Meta-Analyses extension for Scoping Reviews.

## 1. Background

Acute purulent skin and soft tissue infections (SSTIs) also referred to as cutaneous abscesses or type II SSTIs [1] are a common global health complaint, accounting for a third of the most common admissions to the emergency department in the developing world, behind cardiac and respiratory complaints [2, 3].

SSTIs are most prevalent in the male working-age population with other risk factors including obesity, smoking, immunosuppression, anatomical areas of heavy hair growth, and a sedentary lifestyle [2]. SSTIs are considered an urgent surgical presentation requiring prompt intervention [4, 5].

SSTIs are generally caused by an invasion of *β*-haemolytic streptococci, *Staphylococcus aureus*, or community-associated methicillin-resistant *Staphylococcus aureus* (CA-MRSA) within the cutaneous layers of the body, generating a localised cycle of pathogen vs immune response [6].

This cycle within the macroenvironment causes localised inflammation, tissue destruction, and a resulting cavity comprised of pus which is a composition of live and expired neutrophils, bacteria, and debris [7, 8]. Symptoms can range wildly between patients and anatomical locations from localised pain to systemic sepsis and even death in the comorbid individual [9, 10].

In England alone, emergency presentations with SSTIs trebled between 1989 and 2004 from 23,884 to 74,447 admissions per 100,000 population [11, 12]. Treatment failure, concomitant with the rise of CA-MRSA, is now a pivotal issue in this patient group and is a direct causative mechanism in the empirical failure of incision and drainage through proliferation of wound beds and persistent cellular damage [13]. Studies from the United States of America (USA) quote a 47%–94% range of CA-MRSA prevalence within the SSTI patient population [14, 15] with a dearth of contemporary research into this aspect in the United Kingdom (UK).

The problem, and thereby the opportunity for research, arises when one considers that, since the Hippocratic era, the technique of incision and drainage has been the dominating preference of clinicians to achieve infective source control in this patient group [16, 17].

Generally, the standard SSTI treatment pathway generates an admittance into a hospital bed, an acute operating demand, and general anaesthetic to facilitate the incision and drainage procedure, followed by an intensive regime of postoperative wound packing to facilitate healing by secondary intent [4, 16].

The driving premise is offered that the formulaic familiarity with this surgical dogma has blinded clinicians and nurses to the intervention's progressive failure. The efficacy of incision and drainage has been exclusively justified by the narrow scope of empirical infective resolution [18]. While this without doubt should be accepted as an essential outcome for any SSTI intervention, the efficiency of incision and drainage continues to wane [18, 19], likely secondary to the epidemic rise of CA-MRSA [14, 15]. Since 2010, there has been a concern that the technique is no longer sufficient within the contemporary treatment population [20]. And yet, evidence suggests that the technique is practiced in over 90% of cases [21]. Furthermore, when one considers beyond empirical outcomes, there is speculation that wider implications of incision and drainage experienced by the patient, clinicians, and National Health Service (NHS) have, up until now, been vastly overlooked. It is argued that workforce demands, institutional resources and, perhaps most importantly, the physical, psychological, and financial challenges imposed upon the recipient of the surgical intervention reveal an undertow of treatment failure [22, 23].

While the surgical practice of incision and drainage has historic connotations for the medically trained clinician, surgically advanced clinical practitioners now have a contemporary role in performing this intervention [24]. One postulates that the increasing concern and speculation about incision and drainage and its wider causative mechanisms have blossomed through the addition of diverse professions and philosophical outlooks now contributing to and exploring this phenomenon [25, 26].

## 2. The Review

### 2.1. Aim

The aim of this study is to provide a focused landscape of the current interventional management pathway and outcomes in purulent skin and soft tissue infections and to understand why incision and drainage have remained practiced without contemplation or challenge.

This scoping review does not aim to answer a specific question, but rather, to provide an overview of the current knowledge in the SSTI phenomenon. This was rationalised due to the speculation that empirical infective resolution of a SSTI exclusively populates the mainstream of this research landscape [15, 19]. The driving force behind this research is to explore beyond the empirical [25]. Seeking to address the postulation, there are unrecognised holistic mechanisms at play, distorting the empirical perception of success, relative to the management of this condition. This acknowledgment raised several objectives to be explored:

1. Why is I&D practiced without contemplation or challenge?
2. What are the interventional alternatives?
3. How is the success of SSTI management defined in the research?
4. Are there any decision-making processes to direct treatment away from I&D?
5. Is there SSTI research acknowledging levels of reality beyond the immediate outcome of infective source control?

It was supposed that there would be a wealth of available research evidence examining variable clinical treatment methods and outcomes for SSTIs and the resulting wounds following intervention. It was further considered that the philosophical stance of critical realism [25], with a supportive underlying nursing philosophy [26] would drive a review of not only the empirical (person) but also the actual (health and nursing) and real (health, nursing, and environmental) affects that current SSTI treatment practices and outcomes generate.

### 2.2. Design

An evidenced and repeatable approach to the scoping review was chosen, as this supports the key aspects of rigor and appraisal as with the systematic review design [27]. The population, concept, context framework tool (PCC) was chosen in line with guidance from the Joanna Briggs Institute (JBI) for conducting scoping reviews [28–30]. The Preferred Reporting Items for Systematic Reviews and Meta-Analyses extension for Scoping Reviews (PRISMA-ScR) [31] was also utilised to demonstrate a repeatable appraisal and synthesis of the available evidence throughout the review.

### 2.3. Search Methods

Keywords were explored to identify an acceptable saturation of any relevant literature. Through the identification of seminal research papers [15, 32, 33], a multitude of keywords with Boolean operators and truncation [34] were tested to identify a focused return of available literature. Following six searches with keywords in multiple orders, the researchers identified an efficient combination, settling on search term seven (Tables1 and 2): Abscess∗ AND (skin infection OR soft tissue infection) AND (Treatment OR therapy) AND (Drainage OR antibiotics OR aspiration OR suction OR negative pressure).

In January 2022, five databases were searched with the addition of three grey literature sources. Repeat searches were concluded in March 2022 and July 2023, ensuring the most up-to-date and relevant information. The only exclusions applied to the search in support of the scoping view methodology were studies expressed in languages other than English and animal studies. The advancing searches were focused towards identifying terms cited within the title, abstract, or subject term dependent upon the options offered through each database and grey literature source (Tables 1 and 2).

There were no chronological restrictions placed upon the return of potential articles for review. This decision was taken as we speculated that there were viable yet under-researched and unadopted alternative SSTI management practices explored in both a historic and contemporary context. Therefore, to provide a competent and complete landscape of SSTI management knowledge in this scoping review, time of publication was not considered a restriction.

When considering the types of studies eligible for review, the critical realist stance supported the inclusion of all possible study types [25]. The PCC tool was utilised as the recommended framework to acknowledge the intended concepts and postulated outcomes of this scoping review process [30] (Table 3).

### 2.4. Search Outcomes

**Figure 1:**
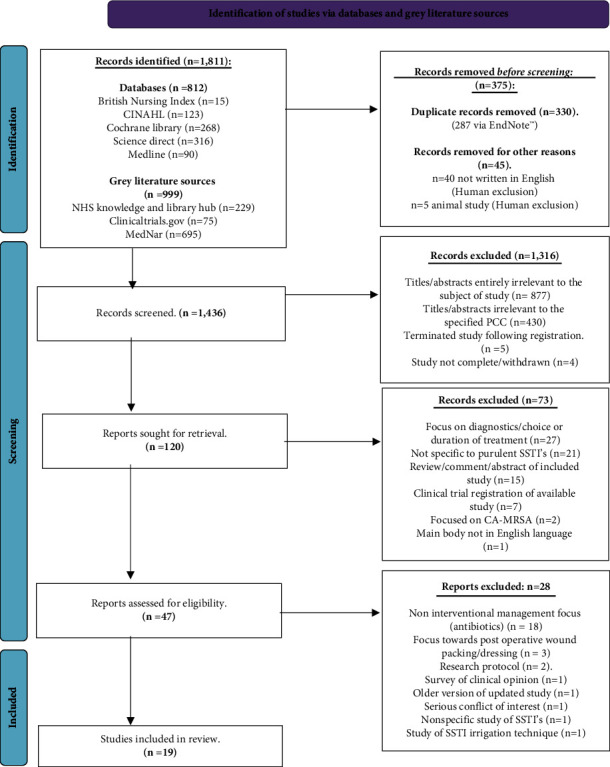
PRISMA-ScR flow diagram [31].

Adopting a systematic approach, the PRISMA tool [31] was used to identify research of relevance (Figure 1). A total of 1,811 results were obtained through all searches across all predefined platforms. The Microsoft™ program EndNote™ was used to correlate the search returns into a designated library. Subgroups were created to correspond with the results from each database. A total of 287 duplicates were removed by the EndNote™ application. A human review of the initial results removed a further 43 duplications and excluded an additional 45 studies due to the predefined exclusion criteria (not written in the English language, *n* = 40; animal studies, *n* = 5). This left 1,428 items available for screening of titles and abstracts.

There were a further 430 papers excluded as the titles or abstracts were found to be at odds with the predefined PCC [30]. There were also nine studies which were removed as they were either terminated (*n* = 5) or withdrawn before completion (*n* = 4). This left 120 papers which were sought for full retrieval and exploration for eligibility.

Of the 120 papers, 27 papers were researching the effects of SSTI diagnostic modality, choice, or duration of treatments in SSTI such as antibiotics, contrasting the PCC [30]. There were 21 studies which turned out to be nonspecific to purulent type II SSTIs or studying complex SSTIs [1]. There were 15 studies which turned out to be personal reviews, commentary on a published study, or abstract/poster references to a published study. Seven papers were in fact clinical trial registrations, two papers were specifically focused on CA-MRSA, and a final study was excluded due to the main body not being written in the English language.

The remaining 47 articles were then screened throughout the full text and assessed for eligibility amongst two authors, with the third available to resolve any generated conflicts.

It was identified that seventeen (*n* = 17) of the fully reviewed papers were specifically focusing on the outcomes of antibiotic treatment following SSTI management [14, 15, 20, 35–48].

Antibiotic therapy is highly researched within the SSTI phenomenon and has become an integral part of contemporary management due to the rise of CA-MRSA [15, 19]. It is, therefore, an important search term to include in this review. However, the driving aim was to acknowledge and landscape the surgical and interventional practices of SSTI management. It was therefore concluded that research explicitly examining the choice of antibiotic therapy postsurgical intervention was unsuitable for final inclusion.

A further four studies were found to be focused towards either the irrigation or dressing of a SSTI wound [3, 49–51] and therefore would not contribute to furthering knowledge relevant to the aims of this review.

Three further returns were found to be clinical trial protocols of studies yet to be undertaken and questionable in their relevance to this review [52–54]. As there was no experimentation or findings to examine, these clinical trial protocols were excluded.

One study was found to be a survey of surgical opinion in traditional SSTI management and therefore would not contribute to new knowledge within this review [55]. A study by Gottlieb and Peksa [56] was identified as an older version of an updated study included in this review [57]. A third study by Long and Gottlieb [58] was found not to be specific in its aims to type II SSTIs [1], and it was felt that any findings from this study could not be generalised to type II SSTI management outcomes nor be utilised in support of any recommendations generated from this review. A final excluded study [59] demonstrated a poor study design with ambiguous results. It is likely that this was due to a serious conflict of interest, in that the primary investigator received a salary and funding from the medical device company whose equipment was used to conduct the study. It was contested that any results from the work of Brody et al. [59] would be tenuous and would add very little to the landscaping of SSTI treatment. With the exclusion of this research, this left final 19 papers to be included within this review. Details of all searches and extractions can be provided by the lead author upon reasonable request.

### 2.5. Quality Appraisal

Although not a stipulation within the framework of a scoping review [60], it was felt necessary to clarify the academic quality of the research papers to be reviewed within the main body of this chapter. This was performed to demonstrate that high-quality rigorous studies had not been favoured in isolation in support of this research, providing a clarity of clinical impact within each research paper examined and informing the reader with a deeper context when summarising the findings of this review. In addition, as this review was being generated in partial fulfilment of a doctoral research qualification, the guidance provided at this level of study demands that a systematic and rigorous appraisal be demonstrated [61].

The Hawker appraisal tool [62] was designed specifically for assessing a wide range of literature from a broad research question of both a quantitative and qualitative nature. When considering the philosophy driving this work, the Hawker tool supports the critical realist perspective to acknowledge all possible levels of reality [25]. The tool seeks to classify each research paper through its relevance to a subject, data extraction, methodological rigor, and findings which are then correlated and expressed numerically out of a possible score of 36 [62]. Whilst credit should be afforded to Hawker et al. [62] for this recognition of healthcare study beyond the empirical, Williams et al. [63] argue that laboriously applying a positivist standard of rigor within a qualitative paradigm is counterproductive, given the polarising epistemologies of the two methods. It could further be argued that a conclusion of trustworthiness within a qualitative study is subjective, generated through the openness of interpretation in the absence of a framework. The Hawker tool addresses this argument by including open-ended, descriptive evaluation in tandem with quantitative dimensions. It was for these reasons that this tool was utilised for the anticipated wide-ranging literature. As per advice from the JBI [28–30], two authors undertook the appraisal process with the third available to resolve any conflicts.

### 2.6. Data Abstraction

The nineteen studies reviewed were almost entirely of an empirical nature, with a focus on the study of SSTI interventional management relative to infective resolution (*n* = 16). Six studies incorporated some quantitative measurements of lived experiences such as pain, daily activities, procedural satisfaction, and experiences with antibiotics [32, 33, 64–67].

The studies were expressed as a collective relative to the geographical focus of study/location, Hawker score [62], intervention, sample size, study design and duration, outcome measurements, and authors conclusions (Table 4). The data was extracted by the lead author and reviewed by the supporting authors as the review progressed.

### 2.7. Synthesis

The identification, categorisation, and expressions of the reviewed studies were performed to generate a narrative relative to the aims and objectives of this scoping review. The synthesis of the evidence was also performed to identify gaps in the current SSTI knowledge.

## 3. Results

Nineteen studies qualified for this review based on the PCC[30]. The studies were conducted within a focus of several geographical locations: *n* = 12 USA [19, 21, 57, 64–66, 68, 70–74], *n* = 2 Turkey [69, 75], *n* = 2 China [33, 77], *n* = 2 International [67, 76], *n* = 1 UK [32]. The studies were also comprised of several methodologies and methods: *n* = 8 cohort studies [32, 64, 65, 68–70, 73, 77], *n* = 5 randomised controlled trials [19, 21, 33, 66, 75], *n* = 2 literature review articles [71, 72], *n* = 2 systematic literature review and meta-analysis [57, 67], *n* = 1 quality improvement study [74], and *n* = 1 meta-analysis [76].

The studies identified ranged in chronology from 1984 [68] to 2021 [57] and studied both adults and children with a simple purulent SSTI [1]. As anticipated, there were several alternative SSTI interventional strategies under scrutiny within the collective literature examined:

1. Traditional incision and drainage [68, 71, 72].
2. Traditional incision and drainage with primary closure [67].
3. Needle aspiration technique [19, 32, 69].
4. Loop drainage technique [21, 57, 65, 66, 70, 73, 75, 76].
5. Modified incision and drainage with indwelling catheter placement [64, 74].
6. Modified incision and drainage with primary closure and suction therapy [33, 77].

**Figure 2:**
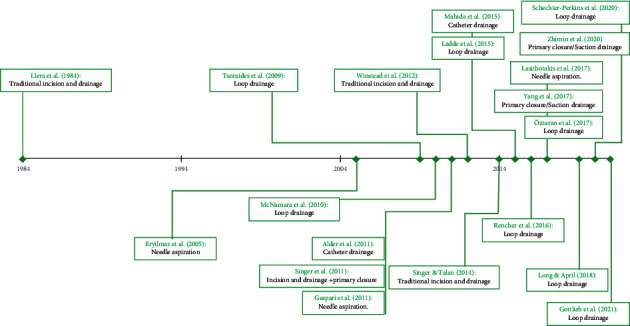
Evolution of SSTI management over time.

Pertinent information synthesised from the literature examined was then developed and expressed in visualisation formats (Tables 4–6; Figure 2).

### 3.1. Efficiency of SSTI Management

The variability of the papers reviewed revealed a comprehensive collection of alternative SSTI management practices with evidenced empirical outcome measurements.

A 75% empirical success rate of the traditional incision and drainage procedure in the resolution of SSTI infection was accepted based on the median historic and contemporary evidence [18, 19, 68, 72]. Taking the median percentile, where available across the relative studies, the empirical efficiency of each SSTI management option can be ranked as follows:

1. Modified incision and drainage with primary closure and suction therapy: 96% [33, 77].
2. Loop drainage: 93.6% [21, 57, 65, 70, 73, 75, 76].
3. Traditional incision and drainage: 75% [68, 72].
4. Traditional incision and drainage with primary closure: 75% [67].
5. Modified incision and drainage with a straight catheter: 75% [64, 74].
6. Needle aspiration: 54.5% [19, 32, 69].

### 3.2. Themes

Across the literature reviewed, the following themes were identified for discussion:

1. Defining SSTIs
2. Defining SSTI treatment failure
3. Patient outcomes
4. Clinician outcomes
5. Nursing outcomes

### 3.3. Defining SSTIs

**Figure 3:**
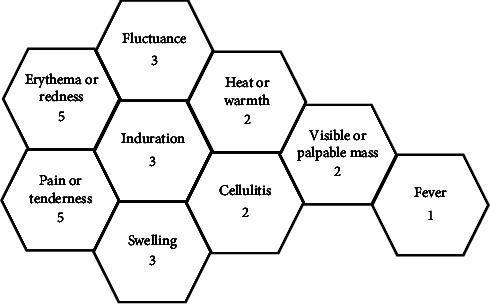
Variables in defining an SSTI.

Throughout the reviewed literature, there were several reoccurring characteristics which were expressed as a diagnostic interpretation of an SSTI.  Figure 3 demonstrates the repeating terms used and how many studies these descriptors were cited in when defining an SSTI.

Interestingly, the presence of a “visible or palpable mass” was one of the least used descriptors, utilised only in the earliest studies reviewed [68, 69]. One could argue that a palpable mass is of unique importance for confirming the presence of a purulent SSTI as opposed to the most used descriptors of “pain” and “induration” which could be seen as rather nonspecific. The term “fluctuance” was a common theme throughout the studies which could be accepted as a clinical indication of purulence, potentially demonstrating an advancement in descriptive terms over time from the generic term “mass”. Of further interest was the use of the terms, “erythema” and “redness” which were frequently used throughout the literature. Whilst one can assume that such presentations are easily observable in lighter skin tones, this has been a speculated causative mechanism in this review, leading to insufficient diagnosis and determination of SSTI progression for individuals with darker skin tones [37, 78]. Finally, although diagnostic criteria were evident throughout this review, twelve out of the nineteen papers made no attempt to define a diagnosis of an SSTI in their studies [21, 32, 57, 64, 65, 67, 70, 71, 73–76].

### 3.4. Defining SSTI Treatment Failure

**Figure 4:**
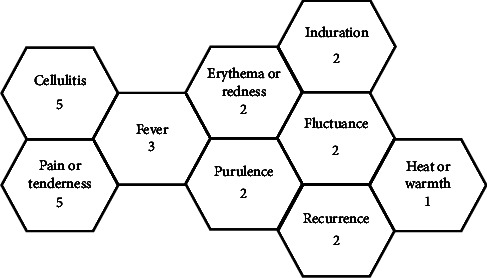
Variables in defining SSTI treatment failure.

This review identified several common themes of descriptive terms used to classify the failure of an SSTI intervention (Figure 4).

One of the least utilised descriptive terms for treatment failure was “SSTI recurrence.” Two research teams [68, 69] classified a repeat SSTI at the original site of intervention or within less than five centimetres of the initial SSTI as a treatment failure. Whilst this may seem sensible, this term lacks the required specifics, like the challenges analysed in the SSTI definition. Interestingly, the most common descriptors used to define treatment failure were the presence of “pain or tenderness” and “cellulitis”. It is countered that these terms could be seen as nonspecific to the recurrence of a purulent SSTI but potentially support the speculation of descriptive progression over time. It is noted that perhaps the most appropriate descriptors of treatment failure would be the presence of “purulence” and “fluctuance”. These descriptors were, however, only used in three of the studies examined [21, 66, 70] with nine further research articles failing to clarify SSTI treatment failure in any capacity [32, 57, 64, 67, 71–74, 77].

### 3.5. Patient Outcomes

It is clear from this review that little credence was afforded to the patient's lived experience, as the recipient of SSTI interventions, and this is a notable theme throughout the review. Although seven of the nineteen included papers did explore some elements of patient experiences such as pain, aesthetics, and use of antibiotics, this was examined in an entirely quantitative capacity [21, 32, 33, 64–67]. Patient experiences formed part of the primary outcome measurements in only one study [33], otherwise demonstrating a scarcity of research recommendations utilising this paradigm in the progression of SSTI research.

Empirical outcomes dominated the design and implementation of the studies reviewed and demonstrated an overwhelming positivist stance towards the resolution of the SSTI infective process [25]. As there is little information available, one can speculate that the priorities of the patient, relative to their SSTI management, may be in stark contrast to those of empirical infective resolution. For example, pain and quality of life may be valued most by recipients of SSTI interventions [23] which are not exclusively paralleled with the empirical focus of the studies synthesised.

### 3.6. Clinical Outcomes

Surgical clinicians made up five of the lead authors across the literature examined throughout this review with the remaining studies led by fifteen physicians and only one nurse (Table 5).

The review has identified a theme that despite a recognised need for new diverse treatment options [64, 72], the evolution of SSTI intervention has been slow to progress due to a persistent culture of clinical resistance. Alternative methods of drainage remain largely unadopted by the surgical community and are a persisting factor in modern research, with 90% of SSTIs still being treated with a traditional incision and drainage technique [21]. When considering why this resistance exists and why almost all SSTI lead researchers have a medical background (95%), the exploration for causative mechanisms and the will to extend knowledge beyond the empirical were severely hampered by a dearth of qualitative or mixed method enquiries. The opinions and values of the surgical team were only acknowledged within one of the studies examined and relative only to an education process during a change to clinical practice [74].

This absence of knowledge often generated a theme of surgical discretion when choosing to undertake a traditional or novel SSTI management practice during research, demonstrating a lack of sociocultural understanding and competent research protocols, outside of randomised controlled trials [64, 70, 74, 75]. When left to the discretion of the surgical clinician, research demonstrated that incision and drainage remained favoured over all treatment methods, despite an available body of evidence [19, 21, 64]. A pertinent demonstration of this was seen in the study by Alder et al. [64] when only 19% of 400 pediatric patients underwent a novel treatment method, having been left to the free choice of an intervening clinician.

### 3.7. Nursing Outcomes

There was only one nurse who took on the role of the lead author in a nonexperimental SSTI research article [71]. When considering the role of supporting author, limited to four of the nineteen studies examined, only four individuals from the nursing profession were acknowledged out of a total of 89 recognised researchers across the literature [21, 64, 70, 71].

As with the theme of patient outcomes, evidenced experiences of nurses treating or managing SSTIs was barren throughout this review. Mahida et al. [74] were the only research team to undertake some form of investigation into the experiences of nurses within this phenomenon, limited to the education of the nursing team during a period of clinical practice change. When challenging historic or ritualistic surgical practices, the limited evidence synthesised indicated that the affected entities of healthcare professionals and institutions should be consulted, educated, and utilised [74]. There was also a complete absence of enquiry into the values and opinions of the nursing team undertaking SSTI management which could help in identifying the nursing and institutional [26] gaps in current management practices during a recognised need for change [21].

## 4. Discussion

While SSTI research has evolved, this has been found to be exclusively in a quantitative capacity. There remains a distinct absence of holistic enquiry despite researchers acknowledging that such studies are required if nurses are to generate new data in the field [32, 64]. This narrow spectrum of SSTI research reflects an overriding realist mentality [25], acknowledging only the empirical aspect of SSTI management and infective resolution. The identified gaps in knowledge generated within this review reveal that empirical outcomes of SSTI management are but a layer of a greater encompassing reality. While empiricism has been the principal focus of SSTI research, the findings of this review have generated a recommendation for a mixed-method or qualitative study, recognising the distinct lack of groundwork previously undertaken when attempting to promote SSTI management innovation.

The findings of this scoping review have revealed that there are many knowledge gaps to address before clinical modernisation in the treatment and management of SSTIs comes to fruition. Without accepting these opportunities, one concedes the likelihood that future research and practice innovation will simply repeat what has gone before, generating the same voids in knowledge and resistance to change that SSTI research needs to explore and address. From the philosophical foundations of nursing [26] and critical realism [25], nurses must now acknowledge and explore evidence-based innovations in SSTI management and utilise the values and opinions of patients, clinical staff, and stakeholders relative to these innovations, addressing the need for qualitative evidence to support alternative SSTI management into clinical practice and professional aceptance. Without these considerations, future SSTI innovation will persist under the theme of empirically constrictive study. Although empirical outcomes are recognised as vital in justifying the efficiency of any SSTI intervention, one counters that we, as nurses, must first acknowledge and direct our innovation in support of the entities directly affected.

From the findings of this scoping review, one could conclude that the healthcare community has a greater understanding of what an SSTI is not, rather than what a SSTI is (Figures 3 and 4). There has been evidence-based focus on the absence of clinical features to determine SSTI resolution rather than actual diagnostic criteria. There remains a varied and sometimes absent consensus within the SSTI research community as to what defines the phenomenon we are exploring. It could be argued further that these variabilities in SSTI description have, collectively, not altered since the first documented incision and drainage procedures in the Hippocratic era [79]. For example, although infrequently used, the terms “erythema” and “redness” appeared to remain a contemporary diagnostic tool in SSTI assessment [21]. Through the critical analysis of the research papers examined [37], one concludes that within today's multiracial societies, less importance should be placed upon these physiological paradigms [78], and we must gain a consensus that supports the entirety of the SSTI patient group.

The apparent clinical resistance to alternative SSTI management practices could be simply a by-product of historical familiarity and the fact that incision and drainage have always been the primary interventions. Therefore, as described by Wallis [80], because we have always done it this way, why should we change our practice? The resistance of the healthcare professionals evidenced within this review is ultimately denying the improvement of patient care and goes against our ethos as nurses and clinicians [26].

The scoping review has yielded evidence that provides founding principles upon which new management pathways can be evidenced for the inclusion of current alternative SSTI management practices. For example, if an objectively healthy patient presents with a pilonidal SSTI, then evidence suggests that needle aspiration with prophylactic antibiotics is a favourable treatment option [32], avoiding the incision and drainage procedure with wound packing. In contrast, there was no evidence found to suggest that needle aspiration would be a suitable option for a patient with a breast abscess [69]. One would therefore consider a more favourable option such as loop drainage [21] or a modified incision and drainage approach with catheter or suction drainage [33, 64]. Evidence also suggests that while an approach using local anaesthetic is a viable option for the adult SSTI population, potentially relieving the institutional demands of historic SSTI practices [25, 26], it is unlikely to be appropriate for pediatric patients [65]. Perhaps surprisingly, the findings from this review suggest that empirical evidence alone does not facilitate a practice change. This recognition has generated some profound unanswered questions which should now be undertaken with clinicians, nurses, patients, and stakeholders to understand why infective resolution and empiricism alone are not driving widespread change in the phenomenon of SSTI treatment and management.

As research continues in its attempts to achieve innovation in SSTI management practices, it is countered, from the perspective of a nurse clinician [26] and a critical realist [25], that the relevant sociocultural groups should be held at the centre of these investigations. Without a deeper context of experiences, one attests that there is no way of understanding the areas of importance and personal value placed upon SSTI management from the required contextual perspectives. For example, infective resolution, ease of use of novel equipment, dexterity, efficiency, training, follow-up demand, and cost effectiveness will all likely play a part in the professional acceptance of alternative SSTIclinical practices. The values and opinions of the patient, however, will likely be in stark contrast and must all be taken into consideration.

If one is to improve upon the interventional management of SSTI patients, it be argued that the act of traditional incision and drainage currently perpetuates failure. There needs to be a definitive change to surgical practice with credence afforded to the unrecognised holistic paradigm in the SSTI phenomenon. It is postulated that only then will contemporary management innovation be achieved and accepted within this field.

### 4.1. Limitations of This Study

The main limitation of this study is in its methodology as a scoping review and the inherent risk of bias generated through this type of review [60]. However, the work was undertaken using a systematic clear approach to minimise a lack of rigor in the study selection, utilising the PRISMA-ScR and PPC tools [30, 31]. Due to our chosen approach to this review, we speculate that additional relevant literature could have been missed due to the predefined search strategy developed by the authors. We further recognise the limitations upon our synthesis of the evidence both as a collective and within the SSTI intervention subgroupings. The ranging methods, aims, objectives, and patient populations used within each study are recognised as a confounding variable in our findings and recommendations.

## Figures and Tables

**Table 1:** Database results.

Sources	Platforms	Search terms	Returned results
Database	Medline (EBSCO host)	Abscess^*∗*^ AND (skin infection OR soft tissue infection) AND (Treatment OR therapy) AND (Drainage OR antibiotics OR aspiration OR suction OR negative pressure)	90

Database	CINAHL (EBSCO host)	Abscess^*∗*^ AND (skin infection OR soft tissue infection) AND (Treatment OR therapy) AND (Drainage OR antibiotics OR aspiration OR suction OR negative pressure)	123

Database	Cochrane Library	Abscess^*∗*^ AND (skin infection OR soft tissue infection) AND (Treatment OR therapy) AND (Drainage OR antibiotics OR aspiration OR suction OR negative pressure)	268

Database	British Nursing Index	Abscess^*∗*^ AND (skin infection OR soft tissue infection) AND (Treatment OR therapy) AND (Drainage OR antibiotics OR aspiration OR suction OR negative pressure)	15

Database	Science Direct	Abscess AND (skin infection OR soft tissue infection) AND (Treatment OR therapy) AND (Drainage OR antibiotics OR aspiration OR negative pressure)	316

Grey literature	NHS knowledge and library hub	Abscess^*∗*^ AND (skin infection OR soft tissue infection) AND (Treatment OR therapy) AND (Drainage OR antibiotics OR aspiration OR suction OR negative pressure)	229

Grey literature	ClinicalTrials.gov	Abscess of skin AND skin infection OR soft tissue infection AND Treatment OR therapy AND Drainage OR antibiotics OR aspiration OR suction OR negative pressure	75

Grey literature	MedNar	Abscess^*∗*^ AND skin infection OR soft tissue infection AND Treatment OR therapy AND Drainage OR antibiotics OR aspiration OR suction OR negative pressure	695

**Table 2:** Final exclusions.

Author and year	Study title	Web link/doi	Comments	Focus of the study	Test intervention reported efficiency in SSTI resolution
Winstead, 2012 [71]	Evaluating and Managing Uncomplicated Skin and Soft Tissue Infections Associated with Community-Associated Methicillin-Resistant *Staphylococcus aureus* for Outpatients: A Review of the Literature	https://doi.org/10.1891/1939-2095.5.2.98	For final inclusion	Traditional I&D	Not Documented

Llera et al., 1984 [68]	Cutaneous Abscesses: Natural History and Management in an Outpatient Facility	https://doi.org/10.1016/0736-4679(84)90002-7	For final inclusion	Traditional I&D	73%

Singer and Talan, 2014 [72]	Management of Skin Abscesses in the Era of Methicillin-Resistant *Staphylococcus aureus*	https://doi.org/10.1056/NEJMra1212788	For final inclusion	Traditional I&D	80%

Alder et al.,, 2011 [64]	A comparison of Traditional Incision and Drainage versus Catheter Drainage of Soft Tissue Abscesses in Children	https://doi.org/10.1016/j.jpedsurg.2011.05.025	For final inclusion	Catheter drainage	75%

Mahida et al. 2015 [74]	Using Quality Improvement Methods to Change Surgical Practice: A Case Example of Pediatric Soft Tissue Abscesses	https://doi.org/10.1097/QMH.0000000000000054	For final inclusion	Catheter drainage	75%

Yang et al., 2017 [33]	A High-Vacuum Wound Drainage System Reduces Pain and Length of Treatment for Pediatric Soft Tissue Abscesses	https://doi.org/10.1007/s00431-016-2835-2	For final inclusion	Suction drainage	96%

Zihmin et al. 2020 [77]	Therapeutic effect of Topical Negative Pressure Therapy/Vacuum-Associated Closure Therapy on Cephalic Facial Skin Abscess	https://doi.org/10.1089/sur.2019.184	For final inclusion	Suction drainage	10–12 days

Tsoraides et al., 2010 [70]	Incision and Loop Drainage: A Minimally Invasive Technique for Subcutaneouse Abscess Management in Children	https://doi.org/10.1016/j.jpedsurg.2009.06.013	For final inclusion	Loop drainage	94.50%

Schechter-Perkins, 2020 [21]	Loop Drainage Is Noninferior to Traditional Incision and Drainage of Cutaneous Abscesses in the Emergency Department	https://doi.org/10.1111/acem.13981	For final inclusion	Loop drainage	88%

Ozturan et al., 2009 [75]	Comparison of Loop and Primary Incision and Drainage Techniques in the Emergency Department	https://doi.org/10.1016/j.ajem.2017.01.036	For final inclusion	Loop drainage	87%

McNamara et al., 2011 [65]	An Alternative to Open Incision and Drainage for Community-Acquired Soft Tissue Abscesses in Children	https://doi.org/10.1016/j.jpedsurg.2010.08.019	For final inclusion	Loop drainage	100%

Gottlieb et al., 2021 [57]	Comparison of the Loop Technique with Incision and Drainage for Skin and Soft Tissue Abscesses: A Systematic Review and Meta-Analysis	https://doi.org/10.1111/acem.14151	For final inclusion	Loop drainage	91.73%

Ladde et al., 2015 [73]	The Loop Technique: A Novel Incision and Drainage Technique in the Treatment of Skin Abscesses in a Pediatric ED	https://doi.org/10.1016/j.ajem.2014.10.014	For final inclusion	Loop drainage	98.60%

Long and Apiril, 2019 [76]	Is Loop Drainage Technique More Effective for Treatment of Soft Tissue Abscess Compared with Conventional Incision and Drainage?	https://doi.org/10.1016/j.annemergmed.2018.02.006	For final inclusion	Loop drainage	95.9%

Rencher et al., 2016 [66]	Comparison of Loop Drainage versus Incision and Drainage for Abscesses in Children	https://doi.org/10.1097/PEC.0000000000001732	For final inclusion	Loop drainage	92.7%

Eryilmaz et al., 2005 [69]	Management of Lactational Breast Abscesses	https://doi.org/10.1016/j.breast.2004.12.001	For final inclusion	Needle aspiration	41%

Gaspari et al., 2011 [19]	A Randomised Controlled Trial of Incision and Drainage versus Ultrasonographically Guided Needle Aspiration for Skin Abscesses and the Effect of Methicillin-Resistant *Staphylococcus aureus*	https://doi.org/10.1016/j.annemergmed.2010.11.021	For final inclusion	Needle aspiration	26%

Lasithiotakis et al., 2018 [32]	Aspiration for Acute Pilonidal Abscess: A Cohort Study	https://doi.org/10.1016/j.jss.2017.09.051	For final inclusion	Needle aspiration	83%

Singer et al., 2011 [67]	Primary Closure of Cutaneous Abscesses: A Systematic Review	https://doi.org/10.1016/j.ajem.2009.10.004	For final inclusion	I&D with primary closure	92.4%

Kotlářová et al., 2021 [42]	Antibiotic Therapy in the Treatment of Skin Abscess Meta-Analysis	https://doi.org/10.33699/PIS.2021.100.7.325-329	Excluded: the effects of antibiotics following traditional I&D		N/A

Talan, 2016 [15]	Trimethoprim-Sulfamethoxazole versus Placebo for Uncomplicated Skin Abscess	https://doi.org/10.1056/NEJMoa1507476	Excluded: the effects of antibiotics following traditional I&D		N/A

Daum et al., 2017 [14]	A Placebo-Controlled Trial of Antibiotics for Smaller Skin Abscesses	https://doi.org/10.1056/NEJMoa1607033	Excluded: the effects of antibiotics following traditional I&D		N/A

Duong et al., 2010 [37]	Randomised, Controlled Trial of Antibiotics in the Management of Community-Acquired Skin Abscesses in the Pediatric Patient	https://doi.org/10.1016/j.annemergmed.2009.03.014	Excluded: the effects of antibiotics following traditional I&D		N/A

Cenizal et al., 2007 [35]	Trimethoprim-Sulfamethoxazole or Doxycycline for Skin and Soft Tissue Infections	https://doi.org/10.1128/aac.00206-07	Excluded: the effects of antibiotics following traditional I&D		N/A

Daum et al. 2016 [36]	Clindamycin versus Trimethoprim-Sulfamethoxazole versus Placebo for Uncomplicated Skin and Soft Tissue Abscesses	https://doi.org/10.1093/ofid/ofw194.111	Excluded: the effects of antibiotics following traditional I&D		N/A

Elliott et al., 2009 [38]	Empiric Antimicrobial Therapy for Pediatric Skin and Soft Tissue Infections in the Era of Methicillin-Resistant *Staphylococcus aureus*	https://doi.org/10.1542/peds.2008-2428	Excluded: the effects of antibiotics following traditional I&D		N/A

Gottlieb, 2017 [40]	Comparison of Trimethoprim-Sulfamethoxazole Versus Placebo for Uncomplicated Skin Abscesses	https://doi.org/10.1017/cem.2016.367	Excluded: the effects of antibiotics following traditional I&D		N/A

Lee et al., 2004 [43]	Management and Outcome of Children with Skin and Soft Tissue Abscesses Caused by Community-Acquired Methicillin-Resistant *Staphylococcus aureus*	https://doi.org/10.1097/01.inf.0000109288.06912.21	Excluded: the effects of antibiotics following traditional I&D		N/A

López et al., 2018 [44]	Comparative Study of Drainage and Antibiotics versus Drainage Only in the Management of Primary Subcutaneous Abscesses	https://doi.org/10.1089/sur.2017.225	Excluded: the effects of antibiotics following traditional I&D		N/A

Powers, 1991 [46]	Soft Tissue Infections in the Emergency Department: The Case for the Use of 'Simple' Antibiotics	https://doi.org/10.1097/00007611-199111000-00005	Excluded: the effects of antibiotics following traditional I&D		N/A

Talan,, 2018 [47]	Subgroup Analysis of Antibiotic Treatment for Skin Abscesses	https://doi.org/10.1016/j.annemergmed.2017.07.483	Excluded: the effects of antibiotics following traditional I&D		N/A

Vermandere et al., 2018 [48]	Antibiotics after Incision and Drainage for Uncomplicated Skin Abscesses: A Clinical Practice Guideline	https://doi.org/10.1136/bmj.k243	Excluded: the effects of antibiotics following traditional I&D		N/A

Fahimi et al., 2015 [39]	The Role of Adjunctive Antibiotics in the Treatment of Skin and Soft Tissue Abscesses: A Systematic Review and Meta-Analysis	https://doi.org/10.1017/cem.2014.52	Excluded: the effects of antibiotics following traditional I&D		N/A

Gottlieb, 2019 [41]	Systemic Antibiotics for the Treatment of Skin and Soft Tissue Abscesses: A Systematic Review and Meta-Analysis	https://doi.org/10.1016/j.annemergmed.2018.02.011	Excluded: the effects of antibiotics following traditional I&D		N/A

Mistry et al., 2014 [45]	Clinical Management of Skin and Soft Tissue Infections in the U.S. Emergency Departments	https://doi.org/10.5811/westjem.2014.4.20583	Excluded: the effects of antibiotics following traditional I&D		N/A

Schmitz et al., 2010 [20]	Randomised Controlled Trial of Trimethoprim-Sulfamethoxazole for Uncomplicated Skin Abscesses in Patients at Risk for Community-Associated Methicillin-Resistant *Staphylococcus aureus* Infection	https://doi.org/10.1016/j.annemergmed.2010.03.002	Excluded: the effects of antibiotics following traditional I&D		N/A

Uknown	Outcomes of a Novel Technique of Mini- Incision and Self-Express (Mise) for Breast Abscess	https://beta.clinicaltrials.gov/study/NCT05762016	Excluded: this is a protocol		N/A

Gulack, 2023 [54]	Conservative Management of Cutaneous Abscess	https://beta.clinicaltrials.gov/study/NCT05461053	Excluded: this is a protocol		N/A

Miller, 2023 [53]	Short- and Long-Term Outcomes of Doxycycline versus Trimethoprim-Sulfamethoxazole for Skin and Soft Tissue Infections Treatment	https://clinicaltrials.gov/show/NCT03637400	Excluded: this is a protocol		N/A

Koehler and Nakayama, 2009 [49]	Treatment of Cutaneous Abscesses without Postoperative Dressing Changes	https://doi.org/10.1016/j.aorn.2009.04.026	Excluded: I&D with or without packing		N/A

Washington University School of Medicine [80]	Abscess Packing versus Wick Placement after Incision and Drainage	https://clinicaltrials.gov/show/NCT01281930	Excluded: I&D with or without packing		N/A

O'Malley et al., 2009 [50]	Routine Packing of Simple Cutaneous Abscesses is Painful and Probably Unnecessary	https://doi.org/10.1111/j.1553-2712.2009.00409.x	Excluded: I&D with/without packing		N/A

Oehme et al., 2020 [3]	Simple Wound Irrigation in the Postoperative Treatment for Surgically Drained Spontaneous Soft Tissue Abscesses: A Prospective, Randomised Controlled Trial	https://doi.org/10.1007/s00268-020-05738-1	Excluded: wound irrigation not intervention		N/A

Rühle, 2021 [55]	International Survey Evaluating Treatment of Primary Superficial Skin Abscesses	https://doi.org/10.1007/s00068-019-01279-y	Excluded: no study of intervention and survey of surgeons		N/A

Brody et al. 2019 [59]	A Novel Silicon Device for the Packing of Cutaneous Abscesses	https://doi.org/10.1016/j.jemermed.2018.12.009	Excluded: serious conflicts of interest		N/A

Gottlieb and Peksa,, 2018 [56]	Comparison of the Loop Technique with Incision and Drainage for Soft Tissue Abscesses: A Systematic Review and Meta-Analysis	https://doi.org/10.1016/j.ajem.2017.09.007	Excluded: older version of updated study		N/A

Long and Gottlieb, 2022 [58]	Diagnosis and Management of Cellulitis and Abscess in the Emergency Department Setting: An Evidence-Based Review	https://doi.org/10.1016/j.jemermed.2021.09.015	Excluded: Not specific to type II SSTIs		N/A

**Table 3:** PCC outcomes.

PCC elements	Definitions
POPULATION	Human participants between the ages of 0–100 with an acute, simple, skin, or soft tissue abscess

CONCEPT	(i) Simple purulent skin and soft tissue infections
	(ii) The incision and drainage surgical intervention and alternative interventional management practices
	(iii) All study methodologies and methods to be considered
	(iv) Empirical treatment outcomes, infection, pain, aesthetics, quality of life, holistic experience

CONTEXT	(i) Nonspecific to region, gender, ethnicity, religion, culture, or sexual orientation
	(ii) English language
	(iii) Hospital and community setting
	(iv) Interventional/surgical management

**Table 4:** General data extraction for scoping review [30].

Author/date/region of study	Hawker score/36	Intervention (s) of study	Study population sample size	Methods	Duration of study	Outcome measurements	Findings
Llera et al. [68], USA	22	Incision and drainage	Adult 78	Observational study	3 months	Patient characteristics, outcomes/complications of incision and drainage	27% recurrence rate following incision and drainage, concluded as the treatment of choice, and study concluded the use of antibiotics were not beneficial in their patient group

Eryilmaz et al. [69], Turkey	26	Needle aspiration vs incision and drainage	Adults 45	Prospective cohort study	3.5 years	Cure rate	Aspiration group failed to demonstrate resolution
						Healing time	Improved healing time with aspiration (*P* =< 0.001)

Tsoraides et al. [70], USA	25	Loop drainage	Children 115	Retrospective cohort study	5 years 9 months	Complications	Successful loop drainage in 94.5% of cases. The mean length of stay 3 days
						Length of stay	

McNamara et al. [65], USA	32	Loop drainage vs incision and drainage	Children 219	Retrospective cohort study	7 months	Complications	Reduced complication rate (0 vs 4 cases)
						Length of stay	Length of stay not significant (*P* = 1.000)
						Wound care, cosmetics, pain	Reduced community wound care. (51.5% vs 0%- *P* =< 000.1)

Alder et al. [64], USA	27	Catheter drainage vs incision and drainage	Children 400	Intention to treat cohort study	1.5 years	Treatment failure	Treatment failure not significant (*P* = 0.188). Significant reduction length of stay (*P* = 0.001). Catheter drainage group required more clinical follow-up (*P* =< 0.001). Significant reduction in wound packing (*P* =< 0.001)
						Complications	
						Length of stay	
						Postoperative wound care, pain, follow-up	

Gaspari et al. [19], USA	29	Needle aspiration vs incision and drainage	Adults 101	Randomised controlled trial	15 months	Treatment failure	Increased treatment failure with needle aspiration (74% vs. 20%)
							47% increased failure rate in patients with CA-MRSA who underwent needle aspiration

Singer et al. [67], international	34	Incision and drainage with primary closure	915 participants across seven studies	Systematic literature review and meta-analysis	Unclear	Time to healing recurrence rates, return to work	Primary closure reduced wound healing time (7.8 days vs 15 days) and allowed for an earlier return to work (4.1 days vs 14.6 days). Similar complication and recurrence rates

Winstead [71], USA	24	Management of uncomplicated skin and soft tissue infections caused by *Staphylococcus aureus*	N/A	Literature review	Unclear	Relevant published literature 2003–2008	Recommendations for incision and drainage only for the treatment of uncomplicated SSTIs in low-risk patients. Incision and drainage combined with antibiotic therapy should be used to manage all high-risk patients

Singer and Talan [72], USA	24	Incision and drainage	N/A	Literature review article	Unclear	Diagnosis, treatment, irrigation, packing, primary vs secondary closure, antibiotics, MRSA	Advocates ultrasound diagnosis, traditional incision and drainage as the mainstay treatment option, routine wound packing unnecessary, alternative practices to be considered in ‘appropriate cases. Limiting antibiotics and wound culture practices

Ladde et al. [73], USA	34	Loop drainage vs incision and drainage	Children 142	Retrospective study	12 months	Treatment failure	Incision and drainage group 17% vs loop drainage group 4% (*P* = 0.03)

Mahida et al. [74], USA	27	Straight drain vs loop drain	Children 681	Intention to treat quality improvement study	2 years 1 month	Uptake of straight drain, treatment failure, outpatient follow-up demand, clinical, and nursing education	78% uptake in favour of straight drainage (*P* = 0.001)
							Nonsignificant decrease in treatment failure *P* = 0.51
							Significant reduction in outpatient follow-up *P* = 0.001

Rencher et al. [66], USA	31	Loop drainage vs incision and drainage	Children 81	Prospective, nonblinded, randomised controlled trial	18 months	Treatment failure, wound appearance, parent satisfaction	Demonstrated noninferiority of the loop drainage technique
							Treatment failure (7.3% loop vs 7.5%)
							Cosmetic appearance at day 14 (6 vs. 6 *P* = 0.43)
							Parent satisfaction rates (86.1% of the loop arm vs 88.2% of the standard arm *P* = 1.00)
							Pain reduction after procedures was similar (*P* = 0.43)

Lasithotakis et al. [32], UK	31	Needle aspiration	Adults 100	Prospective cohort study	4 years	Treatment failure	Successful aspiration in 83% of patient group
						Pain, aesthetics, procedural satisfaction	High level of aesthetic satisfaction (9/10)
							Improved pain postaspiration (9/10 to 5.5/10)

Özturan et al. [75], Turkey	28	Loop drainage vs incision and drainage	Adults 46	Randomised controlled trial	1 year 10 months	SSTI resolution	Resolution rate not significant (*P* = 0.090)
						Adverse events aesthetics, antibiotics	Nonsignificance in secondary outcomes

Yang et al. [33], China	33	Suction drainage vs incision and drainage	Children 1430	Randomised controlled trial	4 years	Pain	Statistically significant reduction in pain *P* < 0.001
						Length of stay	No statistically significant difference in length of stay
						Treatment failure	Significant improvement in treatment time to resolution *P* =< 0.001
						Wound care, pain	

Long and April [76], international	19	Loop drainage vs incision and drainage	460 participants across four studies	Meta-analysis	Unclear	Treatment failure	Incision and drainage failed in 9.43% of cases of cases compared with the loop drainage technique in 4.10% of cases

Schechter-Perkins et al. [21], USA	32	Loop drainage vs incision and drainage	Adults and children 238	Randomised controlled trial	3 years 7 months	Clinical resolution complications, antibiotics	Clinical resolution not significant *P* =< 0.0035
							Reduced additional emergency department attendances
							(1.3 days vs 1.8 days)
							Lower complication rate (9.3% vs. 24.6%)
							Significant reduction in antibiotic requirements (1.3% vs 12.3% (*P* = 0.01))

Zhimin et al. [77], China	23	Suction drainage vs incision and drainage	Adults 47	Cohort study	1 year	Wound healing time	Statistically significant in wound healing time *P* < 0.05
						Recurrence	No statistical significance in abscess recurrence
						Wound care	Reduced number of wound care requirements *P* =< 0.05

Gottlieb [57], USA	34	Loop drainage vs incision and drainage	910 participants across eight studies	Systematic review and meta-analysis	Unclear	Treatment failure	Incision and drainage group 14.7% vs loop drainage group 8.27% (95% CI)

**Table 5:** Authors' professions in SSTI research over time.

Year	Retrieved studies	Profession of lead author (s)	Profession of supporting author (s)
1984	Llera et al. [68]	Emergency physician x1	Physician x1
			Microbiologist x1

2005	Eryilmaz et al. [69]	Surgeon x1	Surgeon x3

2009	Tsoraides et al. [70]	Surgeon x1	Surgeon x1
			Paediatric surgeon x2
			Nurse x1

2010	McNamara et al. [65]	Surgeon x1	Surgeon x2
			Paediatric surgeon x3

2011	Alder et al. [64]	Paediatric surgeon x1	Paediatric surgeon x3
			Nurse practitioner x1
			Nurse x1

2011	Gaspari et al. [19]	Emergency physician x1	Emergency physician x4

2011	Singer et al. [67]	Emergency physician x1	Associate professor of emergency medicine x1
			Professor of emergency medicine x1
			Emergency physician x2

2012	Winstead [71]	Nurse x1	No supporting authors

2014	Singer and Talan [72]	Emergency physician x2	No supporting authors

2015	Ladde et al. [73]	Emergency physician x1	Emergency physician x2
			Research physician x1

2015	Mahida et al. [74]	Surgeon x1	Surgeon x5
			Emergency physician x1
			Research scientist x1

2016	Rencher et al. [66]	Paediatric physician x1	Emergency physician x2
			Paediatric physician x1

2017	Yang et al. [33]	Paediatric surgeon x1	Paediatric surgery team (not otherwise described) x4
			Professor (not otherwise described) x1
			Physician x2
			Research assistant x1

2017	Özturan et al. [75]	Emergency physician x1	Emergency physician x6

2017	Lasithotakis et al. [32]	Surgeon x1	Surgeon x3
			Emergency physician x1

2019	Long and April [76]	Emergency physician x2	No supporting authors

2020	Schechter-Perkins et al. [21]	Emergency physician x1	Emergency physician x1
			Medical physician x1
			Nurse x1
			Research professor x1
			Unknown x3

2020	Zhimin et al. [77]	Emergency physician x1	Emergency physician x1
			Unknown x3

2021	Gottlieb et al. [57]	Emergency physician x1	Emergency physician x2

**Table 6:** SSTI definitions over time.

Author (s)	Definition of an acute SSTI	Definition of SSTI treatment failure
Llera et al. [68]	“Heat (calor), pain (dolor), redness (rubor), and swelling (tumor)”	Return of SSTI in the same anatomical location within 12 months
Eryilmaz et al. [69]	“Redness, warmth, tenderness, induration, palpable mass”	SSTI recurrence
Tsoraides et al. [70]	Not described	Continuing cellulitis and purulent drainage
McNamara et al. [65]	Not described	Fever, cellulitis, pain
Gaspari et al. [19]	Superficial, fluctuance, and induration	Sonographic and clinical variables, not otherwise described
Alder et al. [64]	Not described	Not described
Singer et al. [67]	Not described	Not described
Winstead [71]	Not described	Not described
Singer and Talan [72]	“A swollen, red, tender, and fluctuant mass, often with surrounding cellulitis”	Not described
Ladde et al. [73]	Not described	Not described
Mahida et al. [74]	Not described	Not described
Rencher et al. [66]	“Erythema, induration or fluctuance, and tenderness”	“Worsening erythema, tenderness, induration, and/or fluctuance or patients demonstrating persistent fever and systemic illness”
Lasithotakis et al. [32]	Not described	Not described
Özturan et al. [75]	Not described	Pain and cellulitis
Yang et al. [33]	“Confirm via ultrasound or fine needle aspiration”, fever, and cellulitis	Pain, fever, and cellulitis
Long and April [76]	Not described	Reduction in cellulitis
Schechter-Perkins et al. [21]	Not described	Erythema, warmth, tenderness, induration, fluctuance, purulence
Zhimin et al. [77]	“Redness, discomfort, swelling, and pain”	Not described
Gottlieb et al. [57]	Not described	Not described

## Data Availability

The data that support the findings of this study are available from the corresponding author upon reasonable request.

## Additional Points

*Contributions to the Global Clinical Community*. (i) Recognising there are empirically and holistically viable alternatives to incision and drainage and the management of purulent skin and soft tissue infections. (ii) Recognising positivist, empirically dominated focus within this phenomenon. (iii) Recommendations for a mixed method or qualitative prospective study.
